# Serial Femtosecond Crystallography Reveals the Role of Water in the One- or Two-Electron Redox Chemistry of Compound I in the Catalytic Cycle of the B-Type Dye-Decolorizing Peroxidase DtpB

**DOI:** 10.1021/acscatal.2c03754

**Published:** 2022-10-18

**Authors:** Marina Lučić, Michael T. Wilson, Takehiko Tosha, Hiroshi Sugimoto, Anastasya Shilova, Danny Axford, Robin L. Owen, Michael A. Hough, Jonathan A. R. Worrall

**Affiliations:** † School of Life Sciences, University of Essex, Wivenhoe Park, Essex, ColchesterCO4 3SQ, U.K.; ‡ Diamond Light Source, Harwell Science and Innovation Campus, Oxfordshire, DidcotOX11 0DE, U.K.; § RIKEN, Spring-8 Center, 1-1-1 Kouto, Sayo, Hyogo679-5148Japan

**Keywords:** heme proteins, Compounds I and II, serial femtosecond X-ray crystallography, solvent kinetic isotope effect, water, kinetics

## Abstract

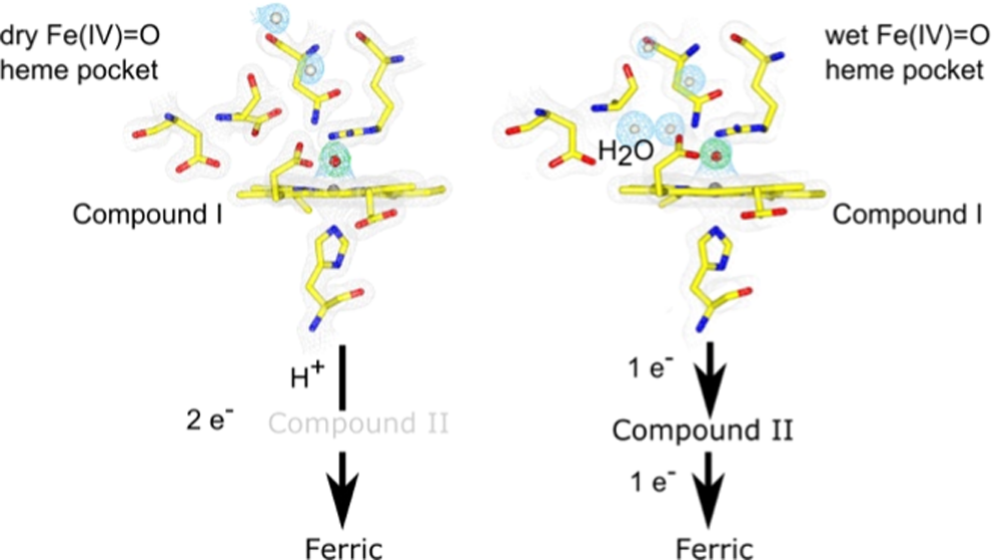

Controlling the reactivity of high-valent Fe(IV)–O catalytic intermediates, Compounds I and II, generated in heme enzymes upon reaction with dioxygen or hydrogen peroxide, is important for function. It has been hypothesized that the presence (wet) or absence (dry) of distal heme pocket water molecules can influence whether Compound I undergoes sequential one-electron additions or a concerted two-electron reduction. To test this hypothesis, we investigate the role of water in the heme distal pocket of a dye-decolorizing peroxidase utilizing a combination of serial femtosecond crystallography and rapid kinetic studies. In a dry distal heme site, Compound I reduction proceeds through a mechanism in which Compound II concentration is low. This reaction shows a strong deuterium isotope effect, indicating that reduction is coupled to proton uptake. The resulting protonated Compound II (Fe(IV)–OH) rapidly reduces to the ferric state, giving the appearance of a two-electron transfer process. In a wet site, reduction of Compound I is faster, has no deuterium effect, and yields highly populated Compound II, which is subsequently reduced to the ferric form. This work provides a definitive experimental test of the hypothesis advanced in the literature that relates sequential or concerted electron transfer to Compound I in wet or dry distal heme sites.

## Introduction

We have tested a long-standing hypothesis that in heme enzymes, a dry distal pocket gives rise to Compound I favoring two-electron redox chemistry, whereas a wet site favors sequential one-electron redox chemistry. Using the same peroxidase scaffold and controlling whether the distal heme pocket is wet or dry, we present experimental evidence to support this hypothesis.

Fe(IV)–oxo complexes, often referred to as ferryl, are the core reactive intermediates found in peroxidases, oxidases, mono, and dioxygenases as well as halogenases and are central to the redox chemistry and reaction products produced by these enzymes.^1,2^ Deciphering the chemical nature of ferryl species, Compound I and Compound II, among heme enzymes has been an intensive area of research.^3−10^ Typically, Compound I consists of an Fe(IV)–oxo species carrying a porphyrin π-cation radical ([(Fe^IV^=O)por•+]),^1,9,11,12^ which can undergo one-electron reduction to Compound II^3^ or two-electron reduction to the ferric state.^13,14^ While there is consensus as to the chemical nature of Compound I across heme enzyme families, the chemical nature of Compound II can vary depending on function. For example, cytochrome P450s possessing proximal Cys–heme ligation have a protonated Fe(IV)–oxo Compound II species (Fe(IV)–OH) under physiological pH.^13,15^ The strong electron-donating ability of the thiolate ligand creates a basic ferryl species, which has the effect of lowering the Compound I reduction potential and suppressing the rate constant for one-electron oxidations of the protein superstructure, e.g., oxidation of Tyr or Trp residues.^13^ This change in the thermodynamic landscape promotes C–H bond cleavage and two-electron oxidation chemistry.^13^ Catalases possess tyrosinate heme ligation, which also affects the basicity of the ferryl species, resulting in Fe(IV)–OH Compound II, enabling two-electron chemistry to disproportionate H_2_O_2_ into oxygen and water.^16^ Thus, a pattern emerges among heme enzymes that possess strongly electron-donating proximal heme ligands that serve to promote a basic ferryl species (p*K*_a_ > 11) and favor reactivity by two-electron chemistries.^13,15,17−20^ Peroxidases on the other hand possess proximal His–heme ligation, which creates a more electrophilic Fe(IV)–oxo species with acidic p*K*_a_ favoring reactions carried out by two sequential one-electron events (Compound I to Compound II (unprotonated) to ferric), typically generating organic substrate-based radicals and also off-pathway protein radicals.^1^

The characterization of basic versus acidic Fe(IV)–oxo species has enhanced our fundamental understanding of how heme enzymes can tune reactivity and function via the nature of the proximal ligand.^2^ However, a recent perspective by Liu^21^ and colleagues recognizes that the nature of the proximal heme ligand may not be as definitive in selecting function. They emphasize that the newly defined proximal His–heme-dependent aromatic oxygenase superfamily (HDAO) can promote oxygen transfer (oxygenase or dioxygenase activity) to aromatic substrates and that other factors such as substrate position within the distal pocket and second sphere coordination can influence reaction outcomes.^21^

Water molecules are often identified in the distal heme pocket of heme enzymes. This led Jones to hypothesize that resident distal heme pocket H_2_O molecules could influence the redox pathway of Compound I, i.e., a wet site would favor a one-electron (peroxidatic) pathway and a dry site would favor a two-electron (catalatic) pathway.^22^ Subsequent computational approaches have provided support that the presence of a H_2_O molecule in the distal pocket lowers the barrier of proton movement from the bound Fe(III)–H_2_O_2_ and facilitates Compound I formation in peroxidases.^23,24^ This has led to the view that peroxidases operate with a wet distal pocket and led to the modification of the original Poulos–Kraut^25^ mechanism of Compound I formation to include a H_2_O molecule that bridges a distal His residue and heme-bound H_2_O_2_.

To test experimentally the role distal heme pocket H_2_O molecules have on the reactivity of the ferric and ferryl species, a system is required whereby the nature of the distal pocket, wet or dry, needs to be unequivocally defined and be amenable to manipulation. Using an X-ray free electron laser (XFEL), we have previously determined room-temperature (RT) serial femtosecond crystallography (SFX) structures of the ferric and Compound I redox states of DtpB,^26^ a B-type member of the dye-decolorizing peroxidase (DyP) family.^27−31^ Such an approach results in “pristine” DtpB structures that do not exhibit the effects of X-ray-generated radiation damage. This is particularly important to metalloenzyme crystals, which are exquisitely sensitive to radiation damage that can result in metal centers being rapidly reduced, potentially instigating structural or solvent positional changes that are then not associated with the starting redox state.^32−34^ The RT-SFX DtpB ferric and Compound I structures revealed that the distal heme pocket, which is composed of an Asp–Arg–Asn triad, was void of H_2_O molecules.^26^ Thus, the H_2_O molecule coproduced upon Compound I formation is not retained. DtpB, therefore, represents an exemplar of a His–heme-ligated peroxidase in which the distal heme pocket favors a dry site. Site-directed mutagenesis has revealed that distal Arg243, and not Asp152, facilitates proton movement on H_2_O_2_ binding to Fe(III)–heme, promoting the heterolysis of the O–O bond.^26^ Therefore, based on Jones’ hypothesis,^22^ Compound I in DtpB would be expected to react via two-electron chemistry.

Herein, we present a series of kinetic and structural experiments that explore the chemistry of both dry and wet distal heme pockets in DtpB and provide evidence to support Jones’ distal heme pocket water theory.^22^ Our results allow us to suggest a novel way in which a His–heme peroxidase can affect two-electron reduction of a substrate.

## Experimental Section

### Site-Directed Mutagenesis

The QuikChange mutagenesis protocol (Stratagene) was used to create site-directed variants of DtpB. The pET28*dtpB* plasmid containing the nucleotide sequence encoding for the wild-type (WT) protein was used as a template to create the N245A variant using the following forward and reverse primers: N245A-F 5′-GAGATCCTGCGGGACGCCATGCCCTTCGGGTC-3′ and N245A-R 5′-GACCCGAAGGGCATGGCGTCCCGCAGGATCTC-3′. For the double, D152A/N245A variant, a pET28*dtpB* plasmid, in which the nucleotides encoding for Asp152 had been changed to encode for Ala, was used together with the N245A forward and reverse primers. To create both mutations, a PCR mix consisting of the respective primers (75 ng μL^–1^), the template (15 ng μL^–1^), 10 mM dNTPs (Fermentas), *Pyrococcus furiosus* (Pfu) Turbo polymerase (Agilent), 10 × Pfu buffer (Agilent), 8% DMSO, and deionized H_2_O was prepared and subjected to the following PCR cycle; 95 °C for 3 min; 16 cycles of 95 °C for 1 min, 62 °C for 1 min (D152A/N245A) or 64 °C for 1 min (N245A) and 72 °C for 8 min; 72 °C for 15 min. Clones were corroborated for the presence of the desired mutation(s) by DNA sequencing (Eurofins).

### Overexpression and Purification of DtpB and Variants

The pET28a (Kan^r^) plasmids containing the desired DNA to overexpress WT DtpB, D152A, R243A, N245A, and D152A/N245A variants were each transformed into *Escherichia coli* BL21 (DE3) cells. Cultures in 2 L shake flasks were grown at 37 °C until an OD_600_ of 0.8 was reached, followed by addition of 5-aminolaevulinic acid (0.25 mM final concentration), iron citrate (100 μM final concentration), and isopropyl β-d-thiogalactopyranoside (IPTG; Melford) to a final concentration of 0.5 mM. The cells were harvested after 16 h following growth at 30 °C, and DtpB and variants were purified as previously reported.^26^

### Preparation of DtpB and Chemicals for Stopped-Flow Absorbance Spectroscopy

Buffers used for stopped-flow kinetic experiments were 50 mM sodium acetate (pH 5.0) and 150 mM NaCl; 20 mM sodium phosphate and 100 mM NaCl (pH 7.0); and a mixed buffer system comprising 10 mM Tris, 10 mM MES, 10 mM MOPS, 10 mM sodium acetate, and 200 mM potassium chloride with the pH adjusted between values of 3 and 10 as required. DtpB and variants were exchanged into the desired buffer using a PD-10 column (Generon) and concentrated using centrifugal ultrafiltration devices (Vivaspin GE Healthcare). The concentration of DtpB and variants was determined by UV–visible spectroscopy (Varian Cary 60 UV–visible spectrophotometer) using an extinction coefficient (ε) at 280 nm of 18 575 M^–1^ cm^–1^. H_2_O_2_ solutions were prepared from a stock (Sigma-Aldrich) with the final concentration determined spectrophotometrically using an ε of 43.6 M^–1^ cm^–1^ at 240 nm. Potassium ferrocyanide (K_4_(Fe(CN)_6_), Sigma-Aldrich) concentrations were determined using an ε of 1046 M^–1^ cm^–1^ at 420 nm. Deuterated buffers were prepared in 99.9% D_2_O (Sigma). Highly concentrated enzymes, K_4_(Fe(CN)_6_), and H_2_O_2_ stocks were diluted directly in D_2_O and left to equilibrate in the D_2_O solutions before experiments.

### Stopped-Flow Absorption Spectroscopy

All transient kinetics were performed using an SX20 stopped-flow spectrophotometer (Applied Photophysics, UK) equipped with a diode array multiwavelength unit and thermostatted to 25 °C. Compound I formation was monitored at various pH/pD ranges: between 3 and 10 for WT, 4 and 10 for D152A, 3 and 9 for N245A, and 4.5 and 10 for D152A/N245A. DtpB and variants (10 μM before mixing) were mixed with a series of H_2_O_2_ or D_2_O_2_ concentrations (ranging from 20 to 1000 μM before mixing), and the overall spectral transitions were monitored. To assess the kinetics of Compound I reduction, K_4_(Fe(CN)_6_) was used at pH/pD values of 5 and 7. Compound I was generated in situ by the addition of one molar equivalent of either H_2_O_2_ or D_2_O_2_ to WT DtpB and variants, before rapidly transferring the syringe to the stopped-flow sample handling unit for mixing with a series of K_4_(Fe(CN)_6_) concentrations (20–10 000 μM before mixing, depending on pH), and the overall spectral transitions were monitored. The analysis of all spectral transitions was performed by fitting the data to selected models in Pro-K software (Applied Photophysics, UK) to yield pseudo-first-order rate constants for Compound I formation and its reduction.

### Microcrystallization of DtpB Variants

Microcrystals of the ferric DtpB variants were grown under batch conditions by mixing in microfuge tubes a 1:1 v/v ratio of a solution containing a 6 mg mL^–1^ DtpB variant in 20 mM sodium phosphate and 300 mM NaCl pH 7 with a precipitant solution consisting of 150 mM MgCl_2_, 150 mM HEPES, and 20% PEG 4000 with the pH adjusted to 7.5 to give a final volume of between 200 and 300 μL. Microcrystals (∼20–100 μm) grew at room temperature within a week.

### Serial Femtosecond Crystallography (SFX)

Due to travel restrictions during the SARS-CoV2 pandemic, data were measured remotely, and therefore, we were unable to use the fixed-target system we have previously used.^26,35^ Consequently, XFEL data were obtained using a high viscosity extruder sample delivery method (see below). The microcrystal suspensions of the ferric DtpB variants were spun in a microcentrifuge (13 000*g*) for 1 min followed by the removal of nearly all of the precipitant solution. The resulting crystal pellet (∼200 μL) was then combined with several other batches to create in total 1–2 mL of a microcrystal slurry with ∼20 μL of the precipitant solution layered across the top of microcrystals. Compound I was generated in the D152A microcrystals by the addition of a stock solution of H_2_O_2_ to give a final concentration of 600 μM. Prior to room-temperature serial femtosecond X-ray (RT-SFX) data collection at the SACLA beamline BL2 EH3, the concentrated microcrystal slurry was filtered using a 30 μm filter and then dispersed into a hydroxyethyl cellulose matrix (HEC)^36^ by mixing 10 μL of microcrystals with 90 μL of 25% (w/v) HEC and homogenized using two syringes connected to each other prior to loading 100 μL into a high-viscosity cartridge-type injector.^37^ The microcrystals in the HEC were extruded at a flow rate of 0.66 μL min^–1^ from a nozzle of 125 μm in diameter into an X-ray beam. The X-ray beam had an energy of 10 keV, a pulse length of 10 fs, beam sizes of 1.39 μm × 1.30 μm for the N245A and D152A/N245A microcrystals and 1.48 μm × 1.10 μm for the D152A, D152A + H_2_O_2_ soak, and R243A microcrystals, and a repetition rate of 30 Hz. We note that the D152A + H_2_O_2_ data set was obtained using a different sample delivery mode (high viscosity extruder) compared to the equivalent WT data that was obtained by fixed-target SFX.^26^ As a consequence, the time delay between the mixing of microcrystals with peroxide and data collection was longer using the extruder. SFX data were processed using the Cheetah pipeline^38^ and CrystFEL 0.10.0^39,40^ with scaling and merging using the Partialator program.

### Structure Determination and Refinement

The SFX structures of the distal heme pocket DtpB variants were solved by an initial refinement cycle using the WT ferric SFX structure (6YRJ) as a model in Refmac5^41^ in the CCP4i2 suite.^42^ The resulting coordinate file was then subjected to model building in Coot^43^ and further refinement cycles. Riding hydrogen atoms and water molecules were added during refinement. No restraints were placed on the Fe–N^ε^His and Fe–O distances. All structures were validated using the Molprobity server,^44^ the JCSG Quality Control Server (https://qc-check.usc.edu), and tools within Coot.^43^ A summary of data collection and refinement statistics is given in Tables S1 and S2, respectively.

## Results and Discussion

### Influence of the Asp–Asn Dyad on the Kinetics of Compound I Formation in DtpB

We have previously reported that the distal heme pocket Arg243 and not Asp152 modulates the kinetics of Compound I formation upon mixing H_2_O_2_ with DtpB.^26^ To complete the kinetic analysis of the Asp–Arg–Asn triad contribution to Compound I formation, the N245A and D152A/N245A variants were created and purified. The electronic absorption spectra and wavelength maxima for the purified triad variants are reported in Figure S1 and Table S3. Using stopped-flow absorption spectroscopy, a single spectral transition was observed on mixing ferric DtpB with H_2_O_2_, consistent with a transition from Fe(III)–heme to a [(Fe^IV^=O)por•+] Compound I species.^26^ A linear dependence of pseudo-first-order rate constants obtained from the global fitting of the spectral data with increasing [H_2_O_2_] was observed, enabling second-order rate constants (*k*_1H_) to be determined (Table 1). For the N245A variant, *k*_1H_ aligns with values for the wild type (WT) and the D152A variant,^26^ with the double variant being an order of magnitude lower (Table 1). As Compound I formation is associated with the breaking and forming of an O–H bond, exchanging the system into D_2_O will inform if these steps are proton rate limited. The experimentally determined second-order rate constant in D_2_O (*k*_1D_) for Compound I formation is reported in Table 1, with the *k*_1H_/*k*_1D_ ratio resulting in a value of ∼1, indicating no solvent kinetic isotope effect (SKIE). Therefore, proton transfer is faster than the binding of H_2_O_2_ to Fe(III)–heme for WT and all variants, and thus, binding is the rate-determining step.

**Table 1:** Second-Order Rate Constants (25 °C) in H_2_O (k_1H_) and D_2_O (k_1D_) at Two pH Values along with Ionization Equilibria (p*K*_a_) for Compound I Formation in WT DtpB and Variants

DtpB	pH 5.0 *k*_1H_(M^–1^s^–1^)	pH 7.0 *k*_1H_(M^–1^s^–1^)	pD 5.0 *k*_1D_(M^–1^s^–1^)	pD 7.0 *k*_1D_(M^–1^s^–1^)	p*K*_a1_	p*K*_a2_
WT	2.7 ± 0.1 × 10^5^	4.7 ± 0.1 × 10^4^	3.0 ± 0.3 × 10^5^	4.2 ± 0.1 × 10^4^	<4.0	6.8 ± 0.2
D152A	1.7 ± 0.1 × 10^5^	8.2 ± 0.2 × 10^4^			5.6 ± 0.1	6.7 ± 0.1
N245A	2.8 ± 0.1 × 10^5^	5.1 ± 0.1 × 10^4^			5.2 ± 0.6	6.4 ± 0.2
D152A/N245A	1.7 ± 0.02 10^4^	2.5 ± 0.1 × 10^4^			6.0 ± 0.7	6.5 ± 1.0

### RT-SFX Structures of the Fe(III)–Heme Distal Pocket Variants

**Figure 1:**
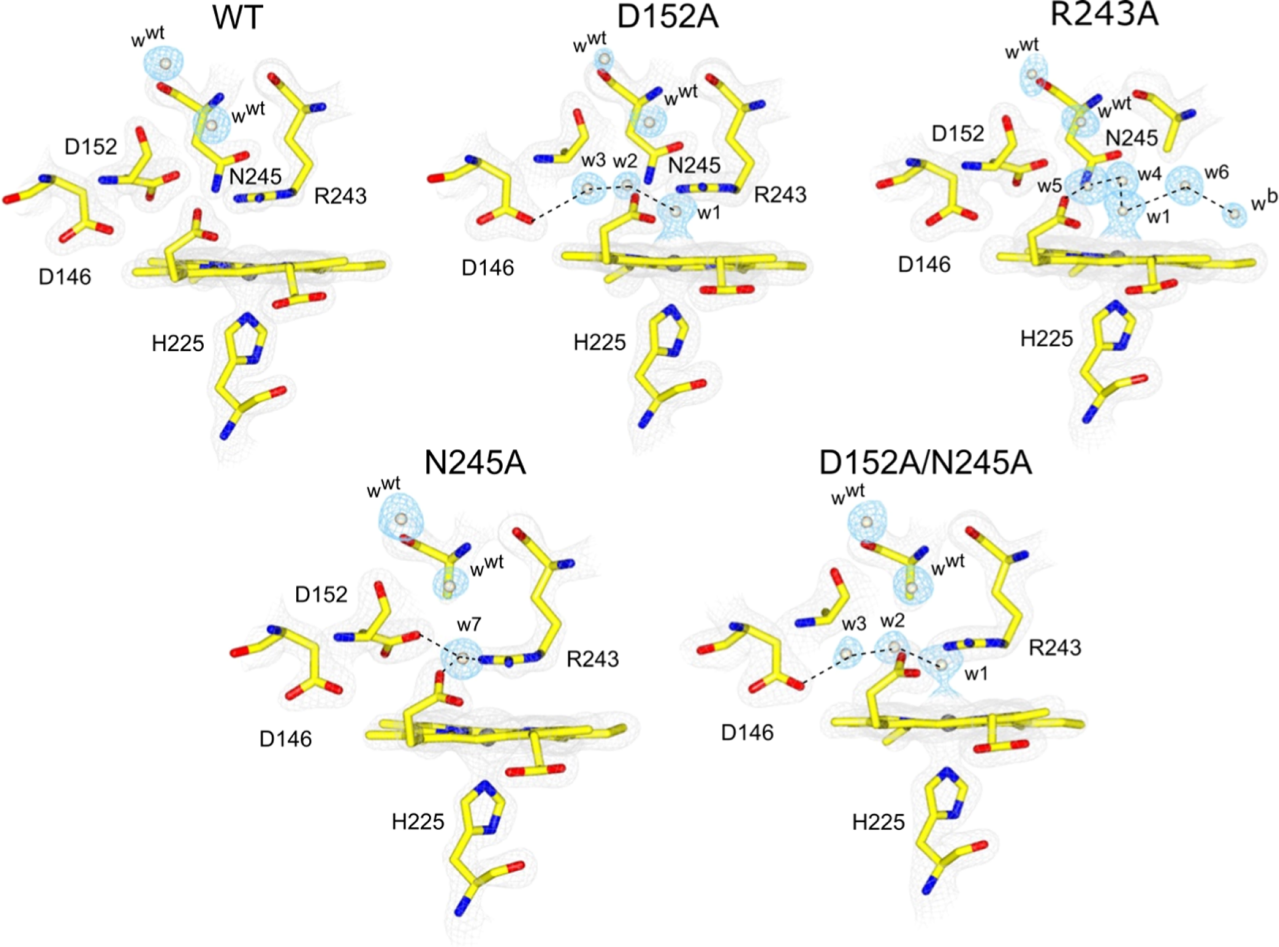
Ferric heme structures of WT DtpB^26^ and variants determined by RT-SFX. 2*F*_o_–*F*_c_ electron density maps for heme and amino acids (gray mesh, contoured between 1.5 and 2.0 σ) and H_2_O molecules (w) in a blue mesh (contoured between 1.0 and 1.7 σ). H_2_O molecules present in the WT structure are labeled w^wt^. H-bonding interactions are indicated by dashed lines.

To assess whether disruption of the Asp–Arg–Asn triad in DtpB leads to a wet distal heme pocket, microcrystals of each variant in the Fe(III)–heme state were produced and subjected to RT-SFX crystallography using a high viscosity extruder sample delivery system^37^ at the SACLA XFEL beamline BL2 EH3. Data collection and refinement statistics for each of the Fe(III)–heme structures are reported in Tables S1 and S2, respectively. For all variants, well-defined electron density peaks were present in the distal heme pocket, consistent with the presence of resident H_2_O molecules (Figure 1). In the D152A variant, three resident H_2_O molecules are accommodated, with two occupying the space left by the Asp152 side chain (Figure 1). In the N245A variant, only one resident H_2_O is found, and in the R243A variant, four resident H_2_O molecules are observed, with three of these accommodated within the distal pocket and the fourth positioned in the space vacated by the Arg243 side chain (Figure 1). This arrangement enables w1 to be directly linked through a H-bonding network to bulk H_2_O (w^b^ in Figure 1). In the D152A/N245A variant, H_2_O molecules are in identical spatial positions to the D152A variant, indicating that in the absence of the Asp–Asn dyad. the preferred spatial arrangement of the resident H_2_O molecules is the space vacated by the Asp152 side chain.

In the D152A, D152A/N245A, and R243 variants, w1 is positioned close enough to the heme–Fe(III) to be a bonding interaction (average Fe(III)–OH_2_ distances within the DtpB hexamer assembly of 2.52, 2.83, and 2.28 Å, respectively (Table S4)). In the N245A variant, the single H-bonded distal pocket H_2_O molecule occupies the space left by the amino group of the Asn245 side chain (Figure 1) but is positioned too far from Fe(III)–heme to initiate a bonding interaction. Thus, Fe(III)–heme in the N245A variant remains pentacoordinate, as in WT DtpB.^26^ Notably, the H-bonded Asp–H_2_O does not have the same spatial stereochemistry as found for the Asp–H_2_O unit in DtpA (an A-type DyP possessing a wet distal heme pocket that uses the Asp–H_2_O unit to facilitate Compound I formation).^10,45^ Thus, the Asp–H_2_O arrangement in the N245A variant would not be expected to facilitate proton movement in Compound I formation. Therefore, we conclude that by disrupting the Asp–Asn dyad, a wet distal heme pocket forms in the Fe(III)–heme state, which does not influence the efficiency of DtpB reacting with H_2_O_2_ (Table 1), indicating that Arg243 facilitates Compound I formation in DtpB regardless of whether the pocket is wet or dry.

### pH Dependency of Compound I Formation

**Figure 2:**
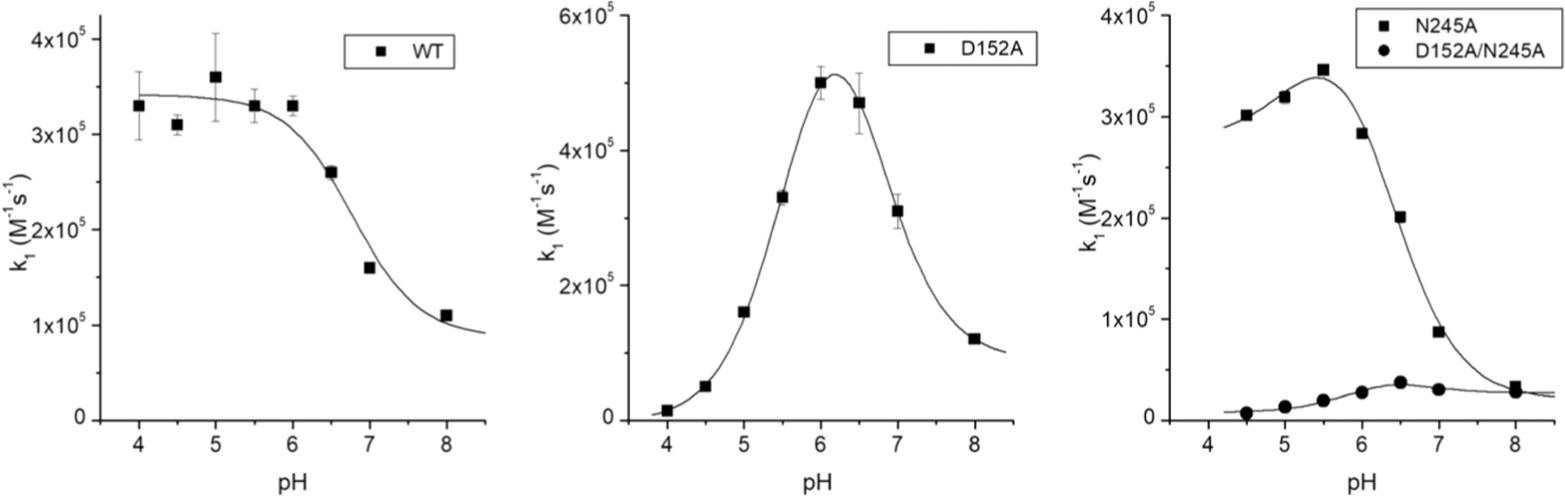
pH dependence profiles for the rate of Compound I formation in DtpB and variants with a fixed concentration (100 μM) of H_2_O_2_ (25 °C). The ionization equilibria constants (p*K*_a1_ and p*K*_a2_) determined from these data are reported in Table 1.

For WT and each variant, the pH profiles of Compound I formation are reported in Figure 2. An obvious feature is that WT DtpB displays a single p*K*_a_, as opposed to the D152A and double variants, which display two p*K*_a_ values (Figure 2 and Table 1). The pH dependence of the N245A variant is closer to that of WT, but the data provide evidence of an acidic p*K*_a_ of < 4 (Figure 2). Structural verification of a wet distal pocket in the Fe(III)–heme variants provides some basis for an explanation of the pH dependency of Compound I kinetics.

We have previously reported for DtpA that the pH dependency of Compound I formation has an acidic p*K*_a_ of ∼4.5, which we assigned to the deprotonation of the bound Fe(III)–H_2_O_2_.^45^ In WT DtpB, we propose that the dry pocket lowers the p*K*_a_ of the bound Fe(III)–H_2_O_2_ and is deprotonated with a p*K*_a_ of < 4. This is a reasonable proposal as moving a positive charge, i.e., a proton, from a dry pocket is energetically favorable. The introduction of H_2_O molecules into the distal heme pocket affects the p*K*_a_ of the bound Fe(III)–H_2_O_2_, by presumably the dipole of the H_2_O molecules partially compensating for the loss of a proton from the bound H_2_O_2_. In the D152A and double variants, H_2_O_2_ would be expected to replace w1, leaving two water molecules in the pocket, both of which participate in an extended H-bonded network (Figure 1), thus supporting a view that H_2_O dipole orientations can compensate for a proton remaining on Fe(III)–H_2_O_2_ and therefore increasing the p*K*_a_ (Table 1).

The N245A variant can be considered to have a “semi-dry” pocket (Figure 1), in which case, the effect of p*K*_a_ on Fe(III)–H_2_O_2_ would be less and is evident inFigure 2, with the p*K*_a_ only partially visualized above pH 4.5. Notably, the pH dependence of the double variant shows that the rates measured are an order of magnitude less than the single variants (Figure 2 and Table 1) while still retaining linear dependencies on [H_2_O_2_]. These results indicate that the on-rate for H_2_O_2_ binding to Fe(III)–heme is much lower in the double variant, but the bound H_2_O_2_ behaves the same as in the single variants. The H_2_O network is identical in the double and D152A variant structures (Figure 1), so the reason for this lower rate constant of H_2_O_2_ binding must be a consequence of removing (or disrupting) the Asp–Asn dyad. The small decrease in the rate of the double variant above pH 6 is unlikely to have the same cause as that of the WT and single variants because the rate of 10^4^ M^–1^ s^–1^ is lower than the lowest rate seen for the WT and single variants at high pH (Figure 2).

Finally, a p*K*_a_ of 6.8 observed in the WT and the two single variants is not influenced by whether the heme pocket is wet or dry, with a deprotonation event influencing the rate limit of Compound I formation. We do not assign this p*K*_a_ to distal Arg243 as it is the deprotonated form of the guanidino group, which facilitates proton movement in Compound I formation.^26^ Instead, we assign the p*K*_a_ to an unknown functional group that on deprotonation gives a negative charge that stabilizes the positive guanidinium form of distal Arg. This makes it more difficult to deprotonate and therefore lower the concentration of the neutral guanidino form, which is essential for the efficient heterolysis of the O–O bond.^26^

### Reduction of Compound I in a Dry Distal Heme Pocket

**Figure 3:**
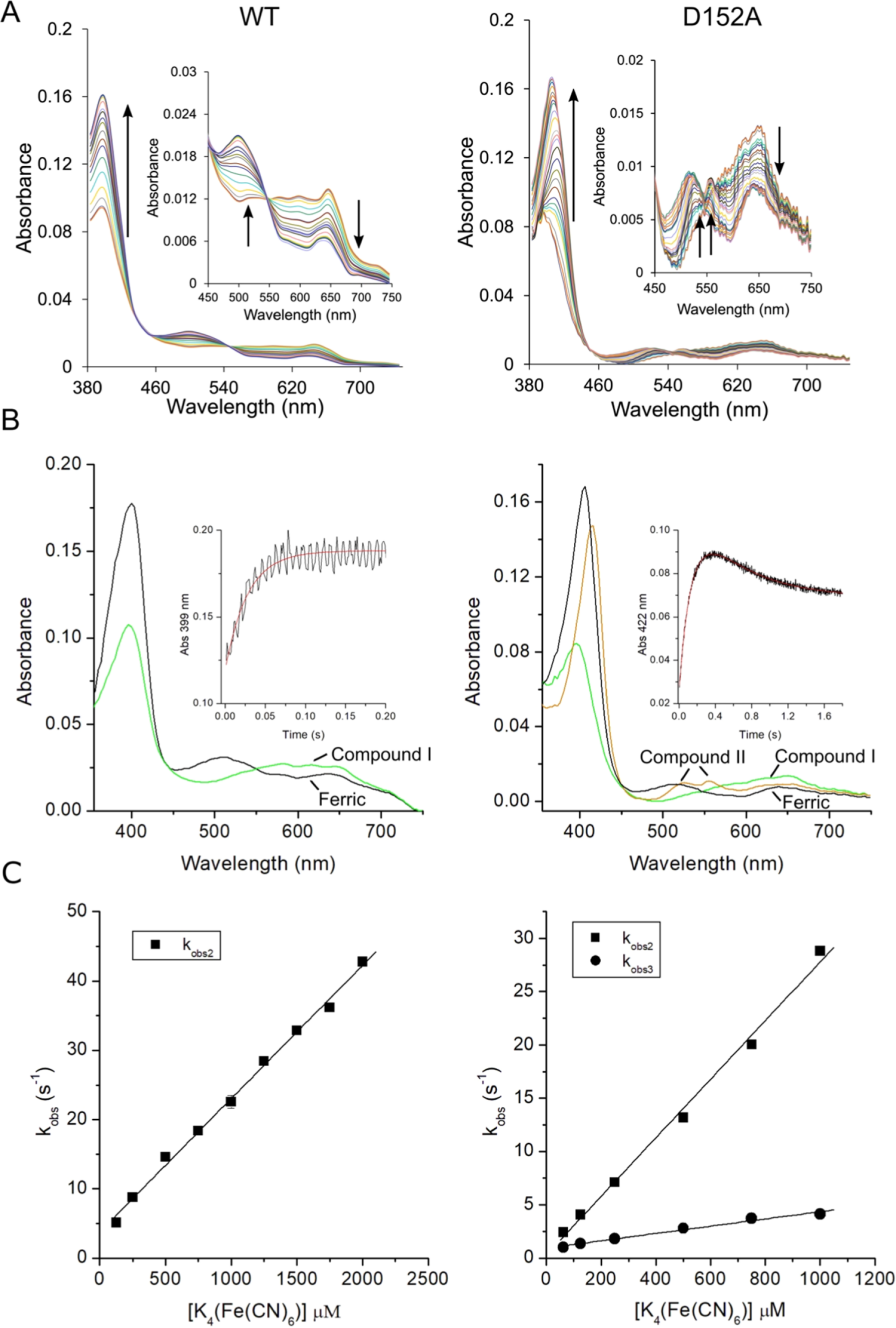
Kinetics of Compound I reduction using K_4_(Fe(CN)_6_) as an electron donor (25 °C, pH 5). (A) Representative time-resolved spectral transitions observed for WT DtpB and the D152A variant upon mixing with 500 and 250 μM K_4_(Fe(CN)_6_), respectively. Spectra were obtained in time ranges of 0–1.2 s for WT and 0–1.8 s for the D152A variant. Arrows indicate the directions of the absorbance changes starting from time zero. (B) Representative global fits of the observed spectral changes for Compound I in WT DtpB and the D152A variant on mixing with 500 and 250 μM K_4_(Fe(CN)_6_). Insets in (B) depict kinetic traces at the specified wavelengths along with their fits (red line) to a single-state transition model (WT) or a two-state transition model (D152A). (C) Pseudo-first-order rate constants *k*_obs2_ (WT), *k*_obs2_, and *k*_obs3_ (D152A) plotted against K_4_(Fe(CN)_6_) concentration, with the solid lines being linear fits to yield second-order rate constants reported in Table 2.

Compound I reduction in a dry distal heme pocket was investigated using K_4_(Fe(CN)_6_) as the electron donor. The green Compound I species generated by stoichiometric addition of H_2_O_2_ shows no spectral decay over 2 h for WT DtpB, allowing for mixing with increasing concentrations of K_4_(Fe(CN)_6_) at pH 5.0 and 7.0 using a stopped-flow absorption spectrophotometer. A single optical transition was observed (Figure 3A) consistent with Compound I being two-electron reduced to the ferric state in a single process with no intermediate being discerned (Figure 3B). Pseudo-first-order rate constants obtained from global fitting of the spectral data (Figure 3B) are linearly dependent on increasing K_4_(Fe(CN)_6_) concentration (Figure 3C), yielding the second-order rate constants (*k*_2H_) reported in Table 2. The transition from Compound I to the ferric state must pass through the one-electron-reduced intermediate, Compound II. However, as the distinct spectral features of this species were not observed at any K_4_(Fe(CN)_6_) concentration, we conclude that *k*_2H_ represents the rate constant for the transition from Compound I to Compound II. Were it otherwise Compound II would be populated and spectrally evident. Based on this and given that the maximum population of Compound II is at most 5% (i.e., at or below our experimental limit), the rate constant for Compound II to ferric must be significantly faster than *k*_2H_. Based on eq 1, which is derived from the Bateman equation^46^ for a three-component sequential reaction (A → B → C)
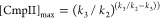
1where [CmpII]_max_ is the fraction of the total concentration of protein maximally present as Compound II. Then, a lower limit for *k*_3_ of 6 × 10^3^ M^–1^s^–1^ can be calculated (where *k*_3_ is the second-order rate constant for the reduction of Compound II to ferric).

**Table 2:** Second-Order Rate Constants (25 °C) in H_2_O (*k*_H_) and D_2_O (*k*_D_) at Two pH Values for the Reduction of Compound I (*k*_2_) and Compound II (*k*_3_) by K_4_(Fe(CN)_6_) in WT DtpB and Variants

DtpB	*k*_2H_(M^–1^s^–1^) pH 5	*k*_2H_(M^–1^s^–1^) pH 7	*k*_3H_(M^–1^s^–1^) pH 5	*k*_3H_(M^–1^s^–1^) pH 7	*k*_2D_(M^–1^s^–1^) pH 7	*k*_3D_(M^–1^s^–1^) pH 7
WT	2.1 ± 0.1 × 10^4^	4.3 ± 0.2 × 10^2^			1.7 ± 0.1 × 10^2^	
D152A	a2.7 ± 0.1 × 10^4^	2.1 ± 0.2 × 10^3^	a3.4 ± 0.2 × 10^3^	4.4 ± 0.6 × 10^2^	2.0 ± 0.1 × 10^3^	3.5 ± 0.1 × 10^2^
N245A	1.3 ± 0.2 × 10^5^	3.9 ± 0.2 × 10^3^	5.6 ± 0.6 × 10^4^	4.5 ± 0.2 × 10^2^	3.1 ± 0.1 × 10^3^	6.0 ± 0.4 × 10^2^
D152A/N245A	a8.5 ± 1.0 × 10^5^	1.2 ± 0.1 × 10^4^	a1.3 ± 0.1 × 10^4^	2.6 ± 0.4 × 10^3^	1.5 ± 0.3 × 10^4^	2.6 ± 0.3 × 10^3^

a Due to instability at pH 5.0, kinetics were carried out at pH 5.8.

**Figure 4:**
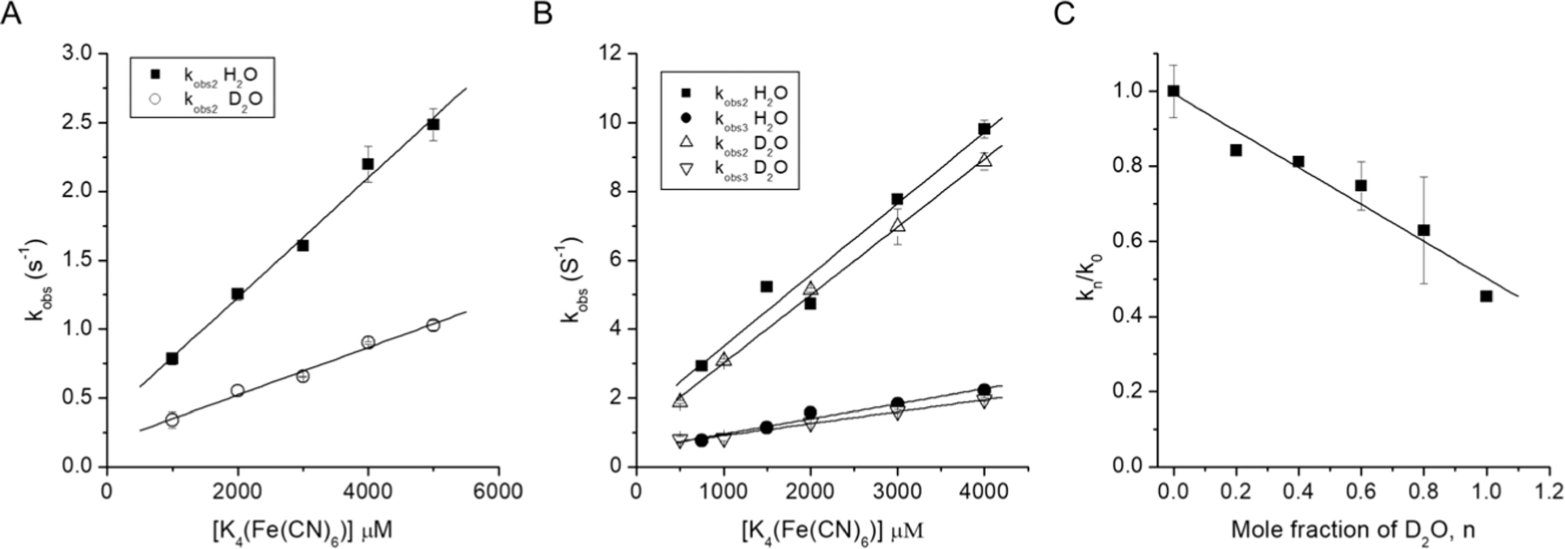
SKIE for Compound I reduction (pD 7.0; 25 °C). (A) Pseudo-first-order rate constants (*k*_obs_) plotted against K_4_(Fe(CN)_6_) concentrations obtained from monitoring the absorbance change of Compound I at 399 nm for WT DtpB (A) and at 422 nm for the D152A variant (B) in H_2_O and D_2_O. The second-order rate constants obtained from linear fits to the data are reported in Table 2. (C) Proton inventory plot for WT DtpB obtained at 5 mM K_4_(Fe(CN)_6_) with *k*_obs_ normalized to 1 in 100% H_2_O in different mole fractions (*n*) of D_2_O, ranging from 0 (100% H_2_O) to 1 (99.9% D_2_O) (*k_n_*/*k*_0_). Data fit best to a linear function, indicative of a one-proton inventory.

In D_2_O, a single optical transition is retained with the second-order rate constant (*k*_2D_) determined at pD 7.0 (Figure 4A) reported in Table 2. The *k*_2H_/*k*_2D_ ratio gives a value of 2.5, consistent with the presence of a SKIE. Determining the *k*_2D_ as a function of the mole fraction (*n*) of D_2_O yields a proton inventory plot (Figure 4C), which reveals a linear dependence of the normalized rate constant (*k_n_*/*k*_0_) against *n*D_2_O, indicating that a single proton is involved in the rate-limiting step. These data imply that the reduction of WT DtpB Compound I is coupled to a proton uptake, and as the rate limit is the transition from Compound I to Compound II, it must be this reaction that is coupled to proton uptake. The reduction of Compound I to Compound II in a peroxidase requires solely the transfer of an electron to the porphyrin ring and does not therefore have a requirement for a proton. However, for DtpB, the transfer of the electron to Compound I is slowed by coupling to proton uptake.

### RT-SFX Structure Determination of the D152A Variant Following Addition of H_2_O_2_

**Figure 5:**
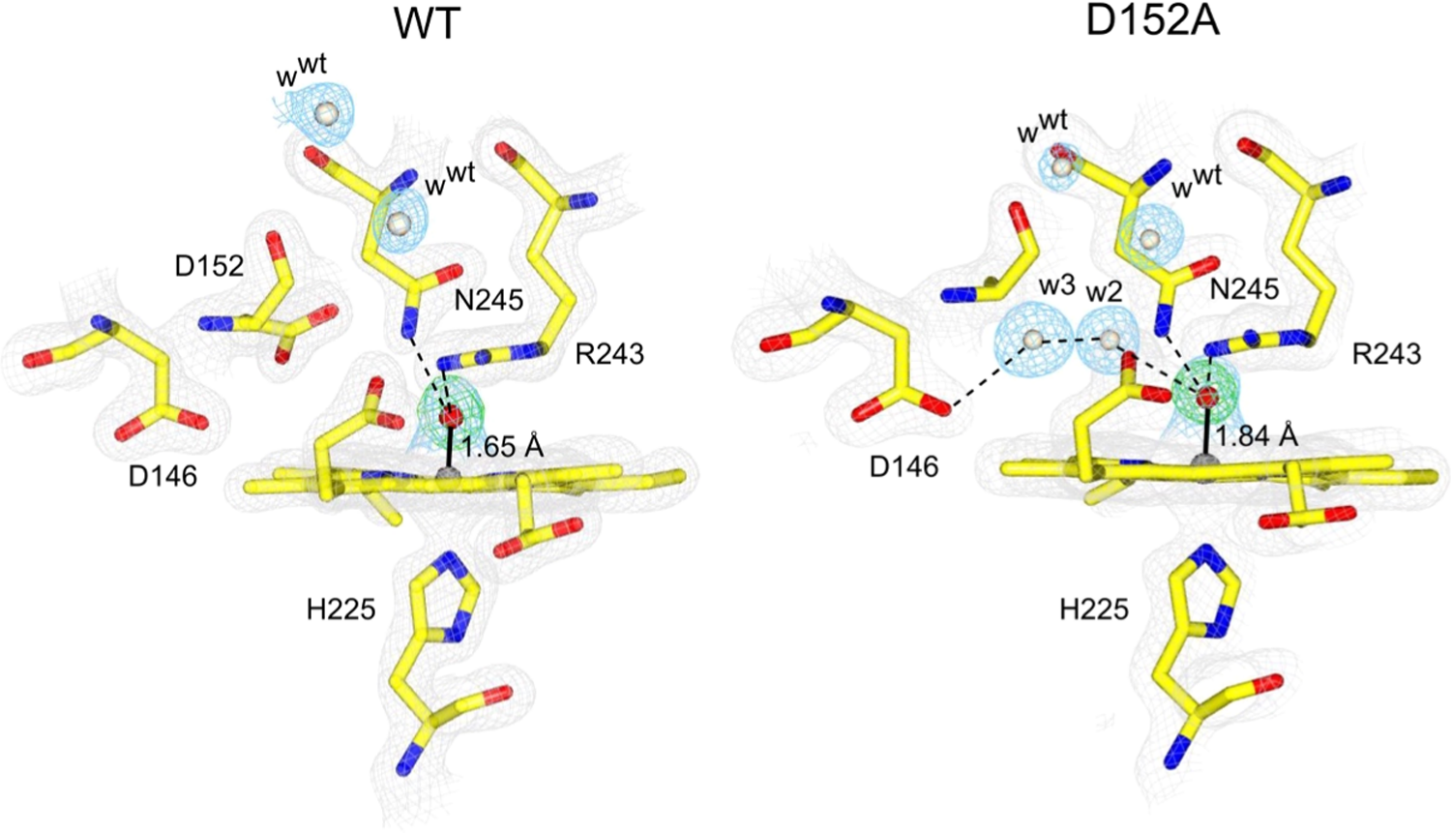
Heme sites of H_2_O_2_-soaked microcrystals of WT DtpB^26^ and the D152A variant determined by RT-SFX. 2*F*_o_–*F*_c_ electron density maps with the heme and amino acids shown in a gray mesh contoured at 2.0 σ (WT) and 1.5 σ (D152A) and H_2_O molecules contoured at 1.0 σ (WT) and 1.4 σ (D152A) in a blue mesh. The *F*_o_–*F*_c_ omit map (green) is shown contoured at ±6 σ (WT) and ±6.7 σ (D152A). This was calculated after a refinement run in which the oxygen atom (red sphere) was omitted from the input model. The Fe-to-O coordination bond is shown as a solid black line and H-bond interactions are indicated with dashed lines.

The stoichiometric addition of H_2_O_2_ to the Asp–Asn variants leads to a green solution with absorption spectra typical of Compound I (Figure S1 and Table S3). However, the lifetime (stability) of Compound I varies among the variants, with the D152A variant displaying no spectral changes for ∼1 h, while for the N245A and D152A/N245A variants, the Compound I spectrum decays toward that of an Fe(III)–heme spectrum within 10–15 min. Previously, we have used a fixed-target chip-based SFX delivery system^35^ to determine the Compound I structure of WT DtpB.^26^ Using this approach, data collection is complete within 20 min (total time following H_2_O_2_ addition 30 min), as opposed to the longer time (>60 min) for the high viscosity extruder system used here. Therefore, based on the D152A variant being the only variant having a Compound I species stable for >15 min, the ferric microcrystals of this variant were subjected to RT-SFX measurement following soaking with H_2_O_2_. Data collection and refinement statistics of the H_2_O_2_-soaked structure determined at 1.90 Å resolution are reported in Tables S1 and S2, respectively. The structure reveals that w2 and w3 remain present, with no new H_2_O molecule observed. An electron density peak is observed directly above the heme–Fe in each monomer of the hexamer assembly. Modeling an O atom into this electron density feature reveals an Fe–O bond length of 1.84 ± 0.15 Å (monomer A; Figure 5), which is significantly shorter than that in the ferric RT-SFX structure, where a H_2_O molecule was modeled with an Fe–O bond length of 2.51 Å (monomer A; Table S4) but longer than the Fe–O bond length (1.65 ± 0.13 Å) in monomer A of WT DtpB following soaking with H_2_O_2_.^26^ As RT-SFX yields an intact structure (i.e., the iron redox state does not become reduced), we are confident to assign the Fe–O species observed in the H_2_O_2_-soaked D152A variant microcrystals as ferryl. However, owing to the longer Fe–O bond length, we are cautious to claim this structure as a “pure” Compound I species, where Fe–O bond lengths of between 1.63 and 1.73 Å are expected.^6,7,47−49^ The longer time scale for the extruder measurements may allow some decay of Compound I (see the kinetics of Compound I reduction below). A recent XFEL study reporting the Compound II structures of yeast cytochrome c peroxidase and ascorbate peroxidase^50^ highlights the possibility of flexibility inherent in Fe–O bond lengths between peroxidase species. However, regardless of whether an Fe(IV)=O or Fe(IV)–OH species is formed, Compound II bond lengths (1.76–1.88 Å)^6,8,14,16,17,47,50−53^ are consistently longer than Compound I bond lengths, and thus, for reasons further discussed below, it could be that the D152A H_2_O_2_-soaked structure is more representative of a Compound II species.

A further point of note is that a wet site creates an additional H-bond donor to the oxo group. The two H_2_O molecules bridge two H-bond acceptors, the Fe(IV)–oxo group and a carboxylate of Asp146 (Figure 5). In synthetic heme Fe(IV)–oxo compounds, H-bond donors (Lewis acids) have been demonstrated to enhance the electron acceptor capabilities of Fe(IV)–oxo, which affects their oxidative and electron transfer properties.^54,55^ Thus, a similar effect on reactivity may be expected in a protein heme pocket.

### Reduction of Compound I in a Wet Distal Heme Pocket

The kinetics of Compound I reduction with K_4_(Fe(CN)_6_) as the electron donor for all DtpB variants were consistent with two phases, (Figure 3A), suggesting the presence of an intermediate species. Global analysis of the full spectral data revealed that the spectrum of the intermediate possessed features consistent with a Compound II species (Figure 3B and Table S3). Thus, unlike WT DtpB, a Compound II species was populated in the variants. The pseudo-first-order rate constants for the reduction of Compound I to Compound II and then to ferric obtained from the global fitting of the spectral transitions revealed linear relationships as a function of K_4_(Fe(CN)_6_) concentration (Figure 3C), with second-order rate constants (*k*_2H_ and *k*_3H_) reported in Table 2. Based on eq 1, the fractional population of Compound II in the variants using the *k*_2H_ and *k*_3H_ values reported in Table 2 may be calculated. At pH 5, there is a clear correlation between the fractional population of Compound II and the “wetness” of the pocket. Thus, the D152A and D152A/N245A variants (fully wet) are almost fully populated at 76 and 93%, respectively, while the “semi-dry” N245A variant is 52% populated. At pH 7, all variants have between 65 and 75% Compound II maximally formed, but the picture correlating with the “wetness” of the pocket seen at pH 5 is now more complex and involves pH dependencies of the individual rate constants. From Table 2, it is further apparent that the reduction of Compound I to II is faster than that seen in WT DtpB and faster than reduction of Compound II to ferric. On repeating the experiments in D_2_O, no SKIE was observed for reduction of either Compound I to Compound II or Compound II to ferric (Figure 4B). Thus, by disrupting the Asp–Asn dyad, the constraint on proton uptake is relieved and Compound I reduction to Compound II is faster than that in the WT DtpB and the SKIE is abolished, i.e., no requirement for proton-coupled electron transfer. In contrast, the rate of Compound II reduction to the ferric state decreases in the variants compared to WT DtpB but is not rate-limited by proton uptake required for H_2_O formation in the Compound II to ferric transition. Therefore, the wet distal pocket in the variants likely provides a ready source of rapidly available protons for this chemistry to occur.

## Conclusions

**Figure 6:**
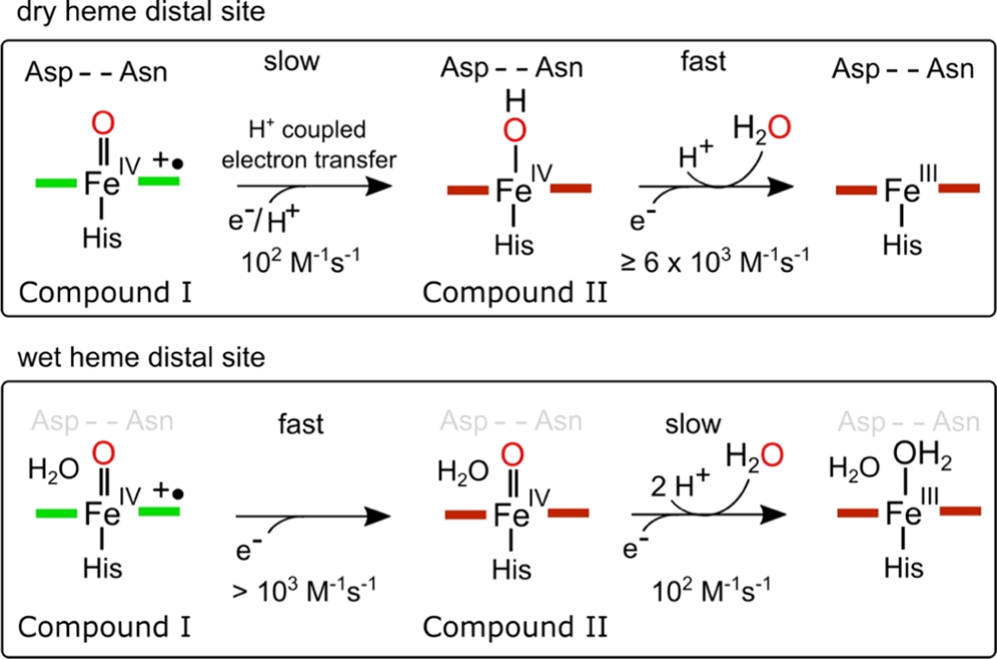
Kinetic mechanism of Compound I reduction in a dry and wet distal heme pocket by disrupting the Asp–Asn dyad. The arrangement of the sequential second-order rate constant in the dry site is consistent with an *apparent* two-electron transfer process from Compound I to ferric, whereas in a wet site, the opposite arrangement of the rate constant explains why two one-electron reactions are observed.

We have assessed through RT-SFX structures and kinetic studies the reactivity of ferric and ferryl heme species in a dry and wet distal heme site within the same peroxidase scaffold. By disrupting the distal heme pocket Asp–Asn–Arg triad in DtpB, our RT-SFX structures reveal that a wet site can be formed. However, disruption of only the Asp–Asn dyad leads to a minimal effect on the kinetics of Compound I formation, which remains dominated by Arg243 (Table 1). Thus, a dry site in DtpB is not the prerequisite to favor Arg over Asp to facilitate Compound I formation. Moreover, a wet site influences the pH dependency of Compound I formation as discussed. A key finding from this work is that the Asp–Asn dyad in DtpB is necessary for the control of proton uptake that accompanies electron transfer to Compound I and enhances greatly the subsequent transition to the ferric form (Figure 6). Our data are consistent with the mechanisms presented in Figure 6, in which the proton coupled to the electron transfer that reduces Compound I in WT DtpB protonates directly the Fe(IV)=O group of Compound II to create a highly reactive Fe(IV)–OH species, that rapidly decays to the ferric state. In a wet site, Compound II is unprotonated and therefore less reactive, leading us to suggest that a difference between wet and dry sites is that the p*K*_a_ of Fe(IV)=O is more basic in a dry site than that in a wet site. Additionally, the long-lived Compound I species in DtpB coincides with the absence of amino acid radicals,^26^ which further suggests that the dry site downwardly tunes the redox potential to diminish the driving force for Compound II formation.^16^ These possibilities serve to highlight that the reactivity/redox chemistry of Compounds I and II in a proximal His–heme-ligated peroxidase can be tuned through the presence or absence of resident H_2_O molecules. Such a conclusion aligns with results with synthetic Fe(IV)–oxo hemes where H-bond donors to the oxo group enhance catalysis.^55^ As the chemistry of Compound I in a dry site is ideally suited to the rapid delivery of two electrons almost simultaneously, this finding serves to support Jones’ hypothesis.^22^ Finally, our findings also provide a useful clue toward identifying the physiological substrates of dry site DyPs. In nature, DtpB can react rapidly with H_2_O_2_ to generate highly stable Compound I, which can await the arrival of ideally a two-electron donor (substrate).

## Supporting Information Available

Data processing and refinement statistics for room-temperature SFX structures (Tables S1 and S2); wavelength absorbance maxima of the ferric, Compound I and Compound II states for the wild type and variants (Table S3); coordinate and hydrogen bond distances at the heme site (Tables S4 and S5); electronic absorbance spectra of the ferric and Compound I states (Figure S1); and stopped-flow reduction of Compound I for the N245A and D152A/N245A variants (Figure S2) (PDF)
