# Supplementary material for: Serial Femtosecond Crystallography Reveals the Role of Water in the One- or Two-Electron Redox Chemistry of Compound I in the Catalytic Cycle of the B-Type Dye-Decolorizing Peroxidase DtpB
Source: ACS Catal. 2022 Oct 18;12(21):13349–59. doi: 10.1021/acscatal.2c03754 (PMC9638988; doi:10.1021/acscatal.2c03754)
Supplement: Supplementary file 1 — cs2c03754_si_001.pdf [file cs2c03754_si_001.pdf]

SUPPORTING INFORMATION

Serial Femtosecond Crystallography Reveals the Role of Water in the One- or Two-Electron Redox Chemistry of Compound I in the Catalytic Cycle of a B-type Dye-Decolorizing Peroxidase DtpB

Marina Lucic,[1] Michael T. Wilson,[1] Takehiko Tosha,[3] Hiroshi Sugimoto,[3] Anastasya Shilova,[2] Danny Axford[2] Robin L. Owen,[2] Michael A. Hough,[1,2] Jonathan A.R. Worrall[1]*

[1]School of Life Sciences, University of Essex, Wivenhoe Park, Colchester, Essex CO4 3SQ (UK). [2]Diamond Light Source, Harwell Science and Innovation Campus, Didcot, Oxfordshire OX11 0DE (UK). [3]RIKEN Spring-8 Center, 1-1-1 Kouto, Sayo, Hyogo 679-5148 (Japan)

*corresponding author: jworrall@essex.ac.uk

EXPERIMENTAL

Table S1: SFX (ambient temperature) X-ray crystallography data processing for DtpB variants in space group $P2_12_12_1$. Values in parenthesis refer to the outermost resolution shell. As is standard practice for SFX data[1], the metrics R_{split} and $CC_{1/2}$ are used to assess data quality and resolution limit in place of conventional crystallographic metrics such as R_{merge} or I/sd(I). The X-ray wavelength was 1.13 Å.

	D152A ferric	N245A ferric	D152A/N243A ferric	R243A ferric	D152A ferryl
Number of merged crystals	29355	10072	18118	13786	27121
Unit cell dimensions (Å)	86.5, 120.9, 197.4	86.9, 121.8, 199.5	86.9, 121.8, 199.5	86.6, 121.3, 197.6	86.5, 120.9, 197.4
Resolution (Å)	36.01-1.92	32.10-2.10	32.10-2.20	35.27-2.00	36.53-1.90
Number of reflections	158842	124109	107809	140951	163211
R_{split}	0.125 (0.887)	0.285 (0.985)	0.219 (1.088)	0.194 (0.790)	0.162 (0.863)
$CC_{1/2}$	0.979 (0.452)	0.878 (0.369)	0.926 (0.509)	0.947 (0.486)	0.968 (0.282)
Multiplicity	606 (427)	627 (469)	1040 (740)	207 (148)	1510 (1172)
Completeness (%)	100 (100)	100 (100)	100 (100)	100 (100)	100 (100)
Wilson B-factor (Å^2)	33.0	32.0	34.0	30.0	31.0

Table S2: Refinement and validation statistics for SFX (room temperature) crystallography for the DtpB variants. Values in parenthesis refer to the outermost resolution shell.

	D152A ferric	N245A ferric	D152A/N243A ferric	R243A ferric	D152A ferryl
Number of reflections	158842	124109	107809	140951	163211
Resolution (Å)	36.01-1.92	32.10-2.10	32.10-2.20	35.27-2.00	36.53-1.90
R_{work}	0.173	0.229	0.218	0.202	0.170
R_{free}	0.202	0.278	0.269	0.230	0.196
RMSD bond lengths (Å)	0.014	0.015	0.016	0.012	0.010
RMSD bond angles (°)	1.767	1.897	1.898	1.653	1.559
Solvent atoms	772	793	687	866	823
ESU based on R_{free} (Å)	0.125	0.212	0.230	0.157	0.118
ESU based on ML (Å)	0.099	0.180	0.191	0.126	0.099
Ramachandran most favoured (%)	97	97	97	97	97
PDB accession code	7QZH	7QZG	7QZF	7ZMJ	7QZE

RESULTS

Table S3: Wavelength absorbance maxima for DtpB and distal heme pocket variants measured at pH 5.0 and pH 7.0 for the D152A/N245A variant, 20 ^{o}C. The ferric and Compound I values were determined using a conventional wavelength scanning absorbance spectrophotometer, whereas the Compound II values are taken from global fitting of the spectral transitions upon mixing Compound I with $K_4[Fe(CN)_6]$ in a stopped-flow spectrophotometer at 25 ^{o}C.

Protein	Ferric-heme (nm)	Compound I (nm)	Compound II (nm)
Wild-type	400, 502, 638	398,577, 612, 648	-
D152A	406, 502, 638	395, 576, 609, 649	416, 525, 555
R243A	404, 502, 625	-	-
N245A	403, 501, 637	398, 575, 613, 649	413, 525, 558
D152A/N245A	405,501, 625	403, 573, 613, 648	413, 525, 552

Table S4: Direct bonding interactions of the ferric heme iron in WT DtpB[2] and the distal heme pocket variants determined from the room temperature SFX structures. Bond lengths to the heme iron in each monomer (chain) of the hexamer assembly as well as the mean distance for the six monomers are reported. The values in parentheses are the mean atomic coordinate error calculated using the DPI server.[3]

Chain	WT	N245A	D152A		D152A/N245A		R243A	
	Fe-N^{ε}His (Å)	Fe-N^{ε}His (Å)	Fe-N^{ε}His (Å)	Fe-H_2O (Å)	Fe-N^{ε}His (Å)	Fe-H_2O (Å)	Fe-N^{ε}His (Å)	Fe-H_2O (Å)
A	2.05	2.09	2.13	2.59	2.17	2.76	2.16	2.23
B	2.10	2.06	2.17	2.55	2.19	2.71	2.16	2.28
C	2.04	2.08	2.16	2.43	2.17	2.85	2.17	2.31
D	2.03	2.07	2.15	2.51	2.15	2.79	2.13	2.28
E	2.02	2.08	2.08	2.54	2.12	3.36	2.12	2.30
F	2.00	2.04	2.13	2.49	2.14	2.48	2.12	2.28
Mean distance	2.04 (0.13)	2.07 (0.30)	2.14 (0.16)	2.52 (0.16)	2.16 (0.34)	2.83 (0.34)	2.14 (0.21)	2.28 (0.21)

Table S5: Coordinate and hydrogen bond lengths for the ferryl heme sites in the SFX structures of WT DtpB[2] and the D152A variant following soaking with H_2O_2. Distances in each monomer (chain) for the distal triad of Asp152, Arg243 and Asn245 to the oxo in the respective ferryl structures are measured from the closest O^{δ} atom of Asp152, the $N^{\eta 1}$ atom of Arg243 and the side-chain amino group of Asn245. The mean distance for the six monomers in the hexamer assembly are reported. The values in parentheses are the mean atomic coordinate error calculated using the DPI server.[3]

Chain	WT					D152A			
	Fe-N^{ε}His (Å)	Fe=O (Å)	Fe=O-Asp (Å)	Fe=O-Arg (Å)	Fe=O-Asn (Å)	Fe-N^{ε}His (Å)	Fe=O (Å)	Fe=O-Arg (Å)	Fe=O-Asn (Å)
A	2.08	1.65	4.39	2.89	2.96	2.13	1.84	2.86	3.05
B	2.13	1.89	4.95	2.83	2.72	2.16	1.96	2.99	2.67
C	2.11	1.89	4.25	2.76	2.67	2.18	2.02	2.96	2.90
D	2.08	1.85	4.28	2.86	2.78	2.14	1.84	2.91	2.92
E	2.06	1.83	4.43	2.79	2.77	2.13	1.99	2.82	2.74
F	2.12	1.80	4.28	2.79	2.80	2.16	1.85	2.98	2.85
Mean distance	2.10 (0.12)	1.82 (0.13)	4.43	2.82	2.78	2.15 (0.16)	1.92 (0.16)	2.92	2.86

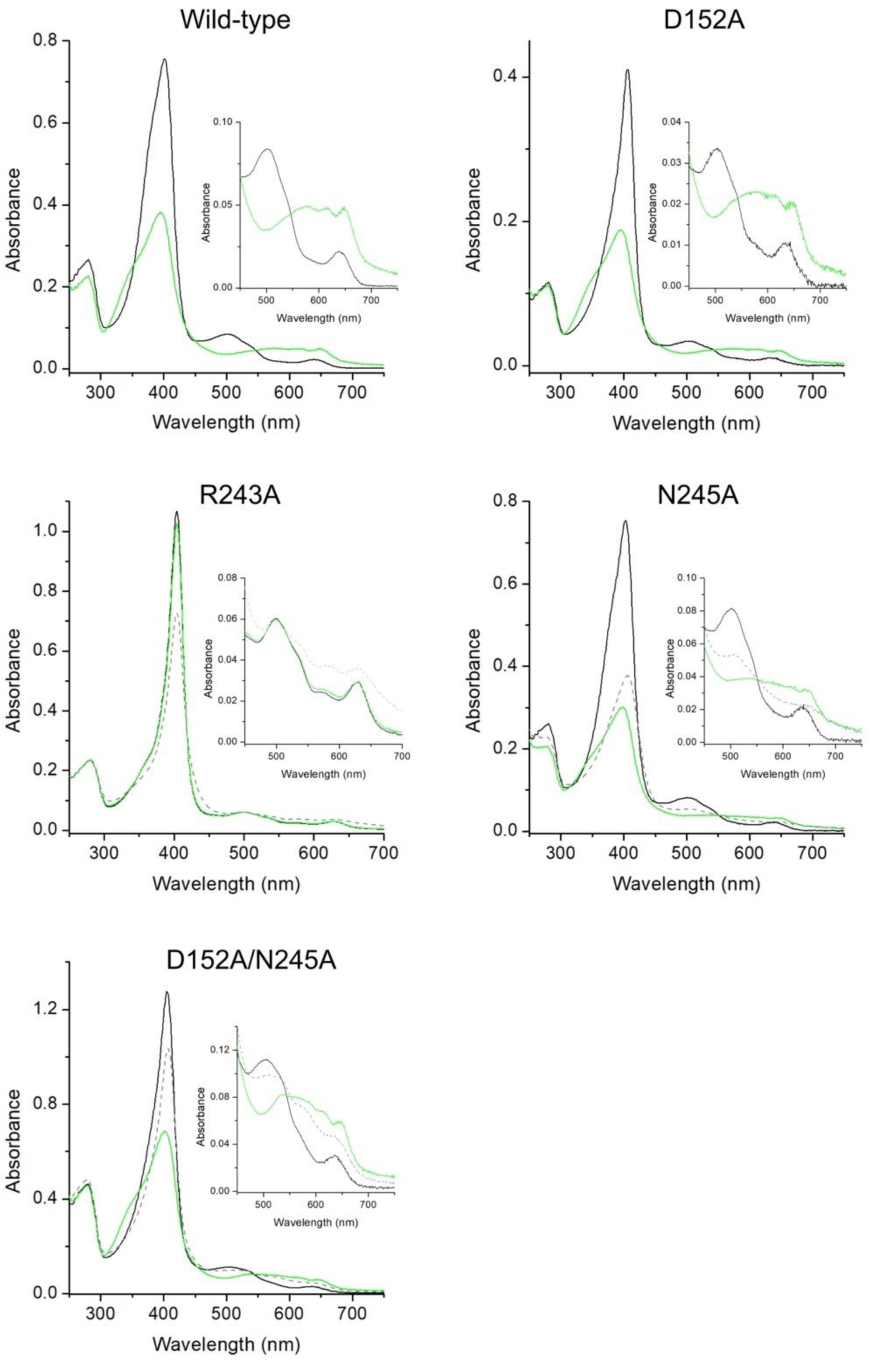

Figure S1: Electronic absorption spectra of DtpB and distal heme pocket variants at 20 °C. Spectra were recorded at pH 5.0 (WT, D152A, R243A, N245A) and pH 7.0 (D152A/N245A). The black line is the ferric heme spectrum, the green line is the spectrum recorded following the addition of a stochiometric molar equivalent of H_2O_2, and where shown the dashed grey line is the spectrum recorded 10 min after addition of H_2O_2. Except for the R243A variant, the green spectra have wavelength features consistent with a Compound I species [(Fe(IV)=O)por•+] and the dashed grey spectra have features consistent with a decay back to

the ferric state. Insets display a magnified Q-band region. Wavelength maxima are reported in Table S3.

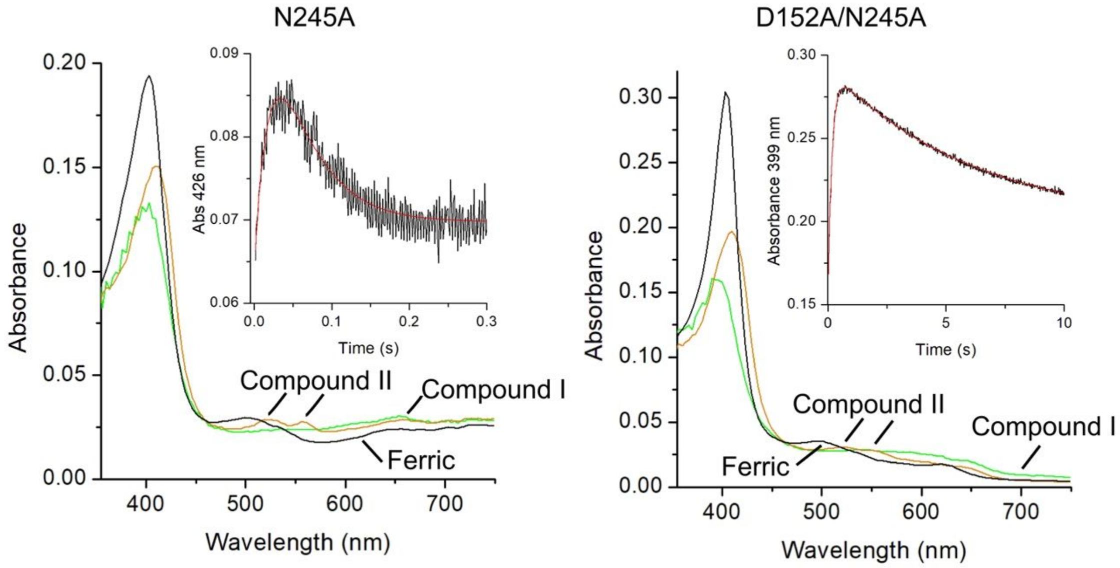

Figure S2: Reduction of Compound I using $K_4(Fe(CN)_6)$ as electron donor. Stopped-flow absorption spectroscopy was used to monitor spectral transitions on mixing $K_4(Fe(CN)_6)$ with the N245A and D152A/N245A variants (25 ^oC). Spectra obtained from global fitting of the observed spectral changes at pH 5.0 (N245A) and pH 7.0 (D152A/N245A), to a model describing a two-state transition. The heme species identified from global analysis are labelled. Inserts depict kinetic traces at the specified wavelengths along with their fits to two-state model (red line). Second-order rate constants, reported in Table 2.

REFERENCES

1. White, T. A.; Kirian, R. A.; Martin, A. V.; Aquila, A.; Nass, K.; Barty, A.; Chapman, H. N., CrystFEL: a software suite for snapshot serial crystallography. *J. Appl. Cryst.* **2012,** *45*, 335-341.

2. Lučić, M.; Svistunenko, D. A.; Wilson, M. T.; Chaplin, A. K.; Davy, B.; Ebrahim, A.; Axford, D.; Tosha, T.; Sugimoto, H.; Owada, S.; Dworkowski, F. S. N.; Tews, I.; Owen, R. L.; Hough, M. A.; Worrall, J. A. R., Serial Femtosecond Zero Dose Crystallography Captures a Water-Free Distal Heme Site in a Dye-Decolorising Peroxidase to Reveal a Catalytic Role for an Arginine in Fe(IV) =O Formation. *Angew. Chem. Int. Ed.* **2020,** *59*, 21656-21662.

3. Kumar, K. S. D.; Gurusaran, M.; Satheesh, S. N.; Radha, P.; Pavithra, S.; Tharshan, K.; Helliwell, J. R.; Sekar, K., Online_DPI: a web server to calculate the diffraction precision index for a protein structure. *J. Appl. Cryst.* **2015,** *48*, 939-942.
